# Breadmaking Quality Parameters of Different Varieties of Triticale Cultivars

**DOI:** 10.3390/foods13111671

**Published:** 2024-05-27

**Authors:** Aliona Ghendov-Mosanu, Nicolae Popa, Sergiu Paiu, Olga Boestean, Viorica Bulgaru, Svetlana Leatamborg, Galina Lupascu, Georgiana Gabriela Codină

**Affiliations:** 1 Faculty of Food Technology, Technical University of Moldova, 9/9 Studentilor St., MD-2045 Chisinau, Moldova; aliona.mosanu@tpa.utm.md (A.G.-M.); sergiu.paiu@doctorat.utm.md (S.P.); olga.boestean@tpa.utm.md (O.B.); viorica.bulgaru@tpa.utm.md (V.B.); 2 Faculty of Food Engineering, “Stefan cel Mare” University, 720229 Suceava, Romania; nicolaepopa1998@gmail.com; 3 Applied Genetics Laboratory, Institute of Genetics, Physiology and Plant Protection, Moldova State University, 20 Padurii St., MD-2002 Chisinau, Moldova; svetlana.leatamborg@sti.usm.md (S.L.); galina.lupascu@sti.usm.md (G.L.)

**Keywords:** bread quality, dough rheological properties, principal component analysis, triticale flour

## Abstract

The aim of this research is to investigate the quality of different triticale cultivars (Ingen 35, Ingen 33, Ingen 93, Ingen 54, Ingen 40, Fanica and Costel) cultivated in the Republic of Moldova from the point of view of the flour, dough, and bread quality characteristics. This research may be of great importance for producers and consumers due to the high production capacity, wide adaptability, economic significance in human foods and nutritional value of triticale cultivars. The triticale flours were analyzed for moisture, ash, protein, wet gluten, fat, carbohydrates, acidity and color parameters (L*, a* and b* values). According to the chemical values, the triticale flours were suitable for breadmaking. The moisture content was less than 14% for all triticale varieties, indicating a long shelf life during its storage and the lowest protein content of 13.1%. The mixing, pasting and fermentation characteristics of triticale dough were analyzed using Mixolab, falling number, dynamic rheometer, alveograph and rheofermentometer devices. All triticale flours presented high levels of α-amylase, with falling number values being less than 70 s. The bread quality characteristics analyzed were the loaf volume, porosity, acidity, and sensory characteristics, and the textural parameters examined were the hardness, gumminess, chewiness, cohesiveness, and resilience. Our data showed large differences in breadmaking quality parameters. However, according to the sensory data, all the bread samples except those obtained from the Costel variety were of a very good quality, being within a total sensory range of 25.26–29.85 points. According to the relationships between flour, dough and bread characteristics obtained through principal component analysis, it may be concluded that the triticale varieties Costel, Ingen 33, Ingen 93 and Fanica, and Ingen 35 were more closely associated with each other. Significant differences were found between the triticale variety samples Ingen 40, Fanica, and Ingen 35 and between Ingen 54, Ingen 33, Costel, and Ingen 93.

## 1. Introduction

Triticale is a hybrid grain created by crossing species of rye (Secale) and wheat (Triticum), which presents the properties of both cereals [1]. According to the information provided by FAOSTAT, from the beginning of the 1990s to the beginning of the 21st century, the cultivated areas of triticale have been continuously increasing [2]. Nowadays, triticale production is around 13 million tons worldwide, and Europe is the major triticale-producing region with almost 90% of the global triticale production and a tendency to expand into areas with soils and climate unfavorable to wheat and rye [2,3]. It is a cereal with a high yield potential; resistance to winter, drought and diseases; tolerance to the toxicity of salts; and high adaptation to the environment (high yields are obtained on slopes, soils, clayey, sandy soils with poor soil) [4,5]. Triticale can easily adapt to different growing conditions, and in this way, it may be a reliable crop for food production. Due to its characteristics, triticale, according to experts, will become one of the leading grain crops in the future [6,7]. Even if it is mostly used as an animal feed, the interest of using it for food production has been increased due to its valuable nutritional composition [8] and health effects [3,6]. Triticale presents a high fiber content (11.7–13.6 g/100 g) and a high amino acid–lysine content (0.31–0.71 g/100 g), which is deficient in wheat grains [6,9]. Compared to wheat, triticale has a higher content of nutrients. Triticale has a comparable starch content (63.3–68.8 g/100 g dry) to rye and wheat [9]. It can produce high levels of α-amylase, which can make the starch more digestible [10]. However, the ratio of amylopectin to amylose can vary considerably. For example, in the case of triticale, the amylose content is very variable, from 12.8 to 35.1 g/100 g of total starch, compared to the amylose content found in wheat, which may vary from 26.9 to 42.8 g/100 g. Regarding the non-starch polysaccharide content, triticale has values much closer to wheat than rye [9,11,12]. The protein content (14–15 g/100 g) may be like those of wheat and rye, but it presents a different amino acid composition. According to Mosse et al. [13], triticale presents intermediate values for serine, leucine, asparagine and aspartate and higher values for arginine and alanine compared to those of parental species. Regarding the content of essential amino acids, triticale is richer in lysine, threonine, tyrosine, tryptophan, methionine and cysteine than wheat and rye [13]. Due to its high nutritional content and the possibility to cultivate it even in difficult conditions, triticale use in breadmaking is of high interest. However, the resistance of triticale to *Fusarium* head blight (FHB) is not well known [14]. According to different authors, it seems that triticale is less resistant to FHB than rye and more resistant than wheat [15,16].

Few studies have been conducted to formulate triticale-based bread in recent years [3,17,18,19]. This may be due to the fact that triticale has a low amount of gluten of poor quality, high α-amylase activity, and a lower dough development time and stability than wheat [18,19]. Among the great variety of triticale grain genotypes, only some are sustainable for bread production. The seven cultivars selected for this study may be part of this group. These are Republic of Moldova cultivars, namely Ingen 35, Ingen 33, Ingen 93, Ingen 54, Ingen 40, Fanica and Costel.

Given the economic significance of triticale in human foods, due to its high production capacity, wide adaptability, nutritional value, and considerable agricultural potential, this study aims to formulate bread products only from triticale flour. For this propose, different rheological, textural and physical–chemical studies have been made. To our knowledge, this is the first study in which triticale dough technological behavior was completely analyzed during mixing, extension, pasting and the fermentation process. Moreover, the significance of the revealed triticale cultivar diversity from the Republic of Moldova is discussed, analyzing their impact for the breadmaking industry. Exploring the possibility of using triticale flour as the main ingredient in breadmaking could increase consumer interest, leading them to seek out bakery products made from other cereal grains than common wheat ones. Indeed, some studies have presented bread formulations using triticale flour, but most of them have used this cultivar as a different percentage addition to wheat flour [3,18,19]. Moreover, dough technological behavior has been partially presented. In this study, to better explore the suitability of triticale in bread, different cultivars have been tested in terms of technological and physico-chemical attributes.

## 2. Materials and Methods

### 2.1. Triticale Flour Samples

The triticale varieties used in the study were Ingen 93, Ingen 33, Ingen 35, Ingen 40, Ingen 54, Costel and Fanica. The grains were cultivated in 2023 at the Institute of Genetics, Physiology and Environmental Protection, State University of Moldova. According to the Chisinau meteorological station data, the average annual air temperature was +12.7 °C and was ranked 1st in years with high average annual temperatures. Additionally, the amount of precipitation in June and July was reduced from 20 to 60 mm.

### 2.2. Physico-Chemical Quality Parameters of Triticale Flour Samples

The triticale grains were ground in a laboratory mill 3100 (Perten Instruments, Hägersten, Sweden), and the following chemical quality parameters of the obtained flours were analyzed according to ICC standard methods: moisture content (ICC 110/1) [20], wet gluten content (ICC 137/1) [20], ash content (104/1) [20], lipid content (ICC 136) [20], and protein content (ICC 105/2) [20]. The acidity value was determined according to Romanian standard SR 90:2007 [21], the carbohydrate content of triticale flour samples was determined by difference: 100–(fat % + protein % + moisture % + ash %), and the color parameters (b*—shade of blue/yellow, L*—darkness/brightness and a*—shade of red/green) were determined using a colorimeter Konica Minolta CR-400 (Tokyo, Japan).

### 2.3. Rheological Properties of Triticale Dough

#### 2.3.1. Rheological Properties of Triticale Dough during Mixing, Pasting and Extension

The triticale dough mixing and pasting properties were determined using the Mixolab device (KPM, Tripette et Renaud, Paris, France) according to the ICC no. 173 standard method [20]. Additionally, the falling number was analyzed according to ICC 107/1 [20] by using a falling number device (FN 1305, Perten Instruments AB, Stockholm, Sweden). The following parameters were determined: water absorption capacity (WA), dough stability (ST), dough development time (DT), torque consistency during stages 1–5 of the Mixolab curve, and the differences between Mixolab torques C12, C23, C34, and C54. Triticale dough extension was analyzed using Alveograph (Chopin Technologies, Villeneuve La Garenne Cedex, France) according to ICC 121 as follows: extensibility (L), swelling index (G), maximum pressure (P), configuration ratio of the Alveograph curve (P/L) and baking strength (W).

#### 2.3.2. Rheological Properties of Triticale Dough during Fermentation

Triticale dough rheological properties during fermentation were analyzed using a rheofermentometer (Rheo F4, Villeneuve-La-Garenne Cedex, France): the total CO_2_ volume production (VT, mL), retention coefficient (CR, %), volume of the gas retained in the dough at the end of the test (VR, mL) and maximum height of gaseous production (H’m, mm). For that, 250 g of triticale flour, 7 g of *Saccharomyces cerevisiae* yeast, and 5 g of salt were mixed, according to the water absorption value determined by the Mixolab test.

#### 2.3.3. Fundamental Rheological Properties of Triticale Dough

The method used for analyzing fundamental triticale dough rheological properties has been described previously by Atudorei et al. [17]. For this purpose, the loss tangent (tan δ) was determined using a HAAKE MARS 40 Rheometer device (Termo-HAAKE, Karlsruhe, Germany) for the frequency sweep tests at a constant stress of 15 Pa.

### 2.4. Breadmaking

The bread was obtained using triticale wheat flour, 3% *Saccharomyces cerevisiae* yeast, 1.5% salt and water according to the water absorption capacity previously determined using the Mixolab device. After dough mixing using a heavy-duty mixer (Kitchen Aid, Whirlpool Corporation, Benton Harbor, MI, USA), the dough was subjected to fermentation at a temperature of 29 ± 1 °C and a relative humidity of 75% in a Cooper 72B fermentation chamber (Europe SRL, Malo (VI), Italy) for 90–120 min according to the fermentation parameters previously established using the rheofermentometer. After fermentation, the dough was divided into 600 ± 1 g rounded pieces, which were then made into rectangular shapes for final fermentation. The final fermentation was carried out in a Cooper 72B fermentation chamber (Europe SRL, Malo (VI), Italy) at 33 ± 1 °C and a relative humidity for 50 min. The dough was steamed and baked using a Cooper 72B fermentation chamber (Europe SRL, Malo (VI), Italy) at 220 ± 2 °C for 50–60 min. The bread was left to cool to 20 ± 1 °C, after which it was subjected to further analysis.

### 2.5. Quality Parameters of Triticale Bread Samples

#### 2.5.1. Physico-Chemical Parameters of Triticale Bread Samples

The triticale bread loaf volume was determined using the seed displacement method. The determination of bread was made using a Fornet device in a medium consisting of rapeseed. Additionally, bread porosity and acidity were analyzed according to the SR 91–2007 standard method [22]. For porosity evaluation, a slice with parallel sides and a height of 60 mm was cut from the middle part of the bread sample. With a standard perforator, previously lubricated with oil, a bread crumb cylinder was obtained. Depending on its volume, density and mass, the porosity of the bread was obtained according to Equation (1):

(1) $$\mathrm{Porosity}\;(\%)=\frac{V-\frac{m}{\rho }}{V}\cdot 100$$

where V is the volume of the crumb cylinder, cm^3^; m is the crumb cylinder mass, g; and ρ is the density of the compact crumb, g/cm^3^. The bread acidity was determine by neutralizing it with 0.1 n NaOH, using phenolphthalein as an indicator.

#### 2.5.2. Textural Parameters of Triticale Bread Samples

The textural analysis was performed using a texture analyzer: the Stable Micro Systems TA.HD plus C, UK. The texture parameters (hardness, gumminess, chewiness, cohesive-ness, and resilience) of the triticale bread samples (40 mm cylindrical probe) were analyzed using the double compression test with a P/75 stainless plate, respecting the following parameters: pretest speed—100 m/s; test speed—5 m/s; post-test speed—5 m/s, and cell load—5 kg [23,24].

#### 2.5.3. Sensory Characteristics of Triticale Bread Samples

The sensory properties of triticale bread samples were analyzed according to ISO 6658:2017 [25] in agreement with the IFST Guidelines for Ethical and Professional Practices for the Sensory Analysis of Foods. A 9-person trained panel of academic staff and food industry experts evaluated the shape and volume; crust appearance and color; baking degree, state and appearance of the bread core; bread core porosity and pore structure; and odor and taste. For all panelists, written informed consent for the sensory evaluation test was obtained, according to the decision of the Technical University of Moldova University Ethics Commission no. 23/21 November 2023. Each sample was tested in duplicate in the sensory laboratory, meeting the requirements of ISO 8589 [26]. The sensory evaluation of the samples was performed using a 30-point scale. Triticale bread samples that received between 24.1 and 30 points were considered very good quality; between 18.1 and 24.0 points were good quality; between 12.1 and 18.0 points were satisfactory quality; between 6.1 and 12.0 were poor quality; and between 0.1 and 6.0 were very poor quality [27,28,29].

### 2.6. Statistical Analysis

The data were analyzed using the analysis of variance (ANOVA) method with Tukey’s test at a significance level of *p* < 0.05. Staturphics software Centurion XVI 16.1.17 (Statgraphics Technologies, Inc., The Plains, VA, USA) was used. To highlight the correlation between data, principal component analysis was performed using XLSTAT 2021.2.1 software (Addinsoft, New York, NY, USA).

## 3. Results and Discussion

### 3.1. Characterization of Triticale Flours

**Figure 1:**
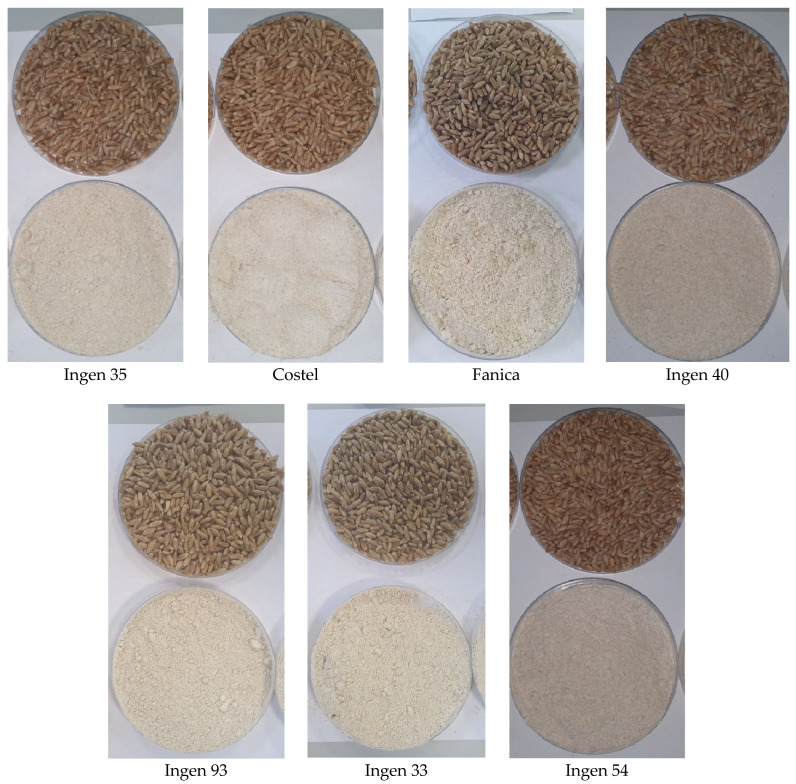
Images of triticale grains and flour varieties used.

The physico-chemical parameters of triticale flours are shown in Table 1, and images of the triticale grains and flours are shown in Figure 1. The flours were obtained using a laboratory mill 3100 (Perten Instruments, Hägersten, Sweden), which produces a homogenous sample required for NIR, glutomatic and falling number analysis of a particle size of approximately 125–150 μm. According to the data content, the moisture content of triticale flours varied between 12.1% and 12.4%, which was less than 14% for all triticale varieties, indicating a long shelf life during its storage [30]. The ash content varied between 1.5 and 1.73%. This is an essential criterion for flour classification and, along with the protein and wet gluten content, a basis for evaluation of the baking value of triticale flour [17]. The protein content varies across all triticale variety flours, the highest amount being recorded to the Ingen 35 variety followed by Ingen 33 and Ingen 93, which has the same protein content of 14.6%. The lowest protein content was obtained for Ingen 40 variety flour, the value of which was 13.1%. This variation in the protein content is in accordance with Alaru et al. [31] due to the triticale variety. Our data on the protein content are lower than those reported by Kaszuba et al. [17] for some Polish and Ukrainian triticale cultivars. The wet gluten content did not present the same trend in terms of the protein content as we expected. A different significance (*p* < 0.05) was obtained between Ingen 93 and Ingen 33, even if these varieties presented the same protein content values. Similar data have also been reported by Kaszuba et al. [17], who also concluded significant differences between the protein content and wet gluten values within triticale flour varieties. The wet gluten content suggests the suitability of using triticale flour from Ingen 35, Ingen 33, Fanica and Costel varieties for breadmaking due to the fact that their values are higher than 22% [30]. However, gluten quality is more important for breadmaking than its quantity. Generally, the fat content significant varies between species, probably due to the triticale variety. This is because fat is located in germ and triticale bran, which may vary from one triticale variety to another. The acidity of the triticale flour is an important parameter, which may affect the quality of the final bakery products, especially from the organoleptic point of view. According to our data, the acidity value of triticale flours varied between 2.5 and 3.9 degrees. This may be related to the rest of the triticale compounds, namely ash, lipid, and protein, which undergo hydrolysis by grain enzymes during fermentation, resulting in the formation of amino acids, fatty acids, or free fatty acids responsible for triticale flour acidity [30]. The color of the triticale flours is generally influenced by the pigments from its composition. It may be given by the white-yellow endosperm particles, due to their content in carotenoid pigments, and by the dark-colored bran particles, given by their flavonic pigments. A loss of brightness (lower L* values) may indicate less flavonic compounds, whereas higher a* values may indicate more red pigments such as xanthophylls [32]. The value of the b* parameter may indicate that the flour has a yellow color, which may be due to the presence of carotenoids from the triticale flour [23].

### 3.2. Triticale Dough Rheological Properties

Triticale dough rheological properties during mixing and pasting are shown in Table 2. The Mixolab device offers a lot of information related to the technological behavior of dough during mixing and heating and even on bread shelf-life quality after baking [33,34]. In the first stage of the Mixolab curve, dough behavior during mixing was provided. According to our data, significant differences (*p* < 0.05) regarding the water absorption (WA) value between triticale varieties have been obtained. This may be due to the different composition of triticale flours. It is known that the two major components of flour, proteins and starch, bind the majority of water in the dough [35]. Additionally, pentosans play an important role in the binding water process in the dough system [36]. Taking into account the fact that the triticale varieties had a protein content that varied significantly (*p* < 0.05) and that this is the most important component, which binds water in the dough system, the variation in this parameter for the triticale samples was somewhat predictable. The dough development time and dough stability are some of the most important parameters for breadmaking. The higher these parameters are, the stronger the flour is for breadmaking and the better quality it is [37]. The highest values of these parameters were recorded for Ingen 40, Ingen 54 and Fanica triticale flour samples. This may be due to lower proteolytic activity from these triticale flours. According to the Mixolab data, the dough obtained from these triticale flour samples presented the highest C2 values and the lowest C12 ones. It is well known that C2 is a measure of protein weakening and the differences between points C1 and C2 (C12) of the protein weakening speed under heating [33]. When the dough temperature began to increase, the dough viscosity decreased, especially due to the proteolytic activity, which reached the optimum activity and broke down the proteins from the dough system, leading to a decrease in C2 and an increase in C12 Mixolab values. The Mixolab C3 torque did not present any significant differences (*p* < 0.05) between Ingen 33, Ingen 93 and Ingen 54 varieties, but significant differences were noted for the rest of the triticale dough cultivars. This behavior may be related to different factors such as water absorption, moisture content and α-amylase values [38]. A similar trend was recorded for the differences between torques C3 and C2 (C32), which is a measure of the starch gelatinization speed [33]. As the starch broke down, the differences between Mixolab torques C3 and C4 increased, which is a measure of amylase activity. It is well known that the falling number value (FN) expresses α-amylase activity. According to our data, lower values of FN lead to lower C34 values, which agrees with those reported by others [38]. However, according to FN values, all triticale flours present low α-amylase activity [39]. The high variability of C3 torque between triticale flours may be due to the fact that its value depends not only on the α-amylase content from the dough system but also on the water absorption and even moisture content of the triticale flours [38]. The C4 torque offers information about hot gel stability. It may be affected by different factors such as the wet gluten content, which may interact with starch decreasing the dough viscosity values [40]. It also may be affected by enzymatic activity, which acted up during the Mixolab stage, causing gluten dehydration or an increase in the maltose amount, which may lead to a decrease in dough viscosity [34]. Lower values of C5 torque and the differences between C5 and C4 indicate lower retrogradation of the starch. According to the data obtained, some triticale varieties, such as Costel, Fanica, Ingen 33, Ingen 93, may lead to bakery samples with a longer shelf life.

By comparing the alveograph data shown in Table 3, it may be seen that all triticale dough samples presented high tenacity and low extensibility values expressed by L and G parameters. However, the baking strength (W) is not very high for any of the triticale dough samples, meaning that none of these flours are strong for breadmaking. These results were in agreement with those reported by others [41]. The variation in the rheological parameters during biaxial extension may be due to the water amount from the dough system. For example, a higher protein content and especially gluten in triticale dough will require a larger amount of water during mixing [42]. However, according to the standard method of alveograph analysis, all analyses have been made at constant hydration. Since the moisture content of the triticale flours was similar, the amount of water added for alveograph analysis was not very different for the biaxial extension analysis. According to the Mixolab data obtained, WA was significantly different (*p* < 0.05) among triticale samples. Adding less water than the optimum amount for dough component hydration can lead to higher viscosity, which may be reflected by an increase in P and W values. As may be seen, the highest values of W and P were recorded for the Fanica variety, who also needed the highest amount of water to be added for optimum dough component hydration according to the Mixolab WA value. However, a high influence on alveograph parameter values may also have been due to the enzymatic activity from the dough system. For example, Ingen 33 and Ingen 54 may present higher proteolytic activity (high C32 values), which may lead to lower alveograph values. Moreover, according to Chavoushi et al. [41], a lower monomeric-to-polymeric gluten protein ratio from triticale flours and higher α-amylase activity significantly affect the alveograph values.

The rheofermentometer values are shown in Table 4. As may be seen, high values of VT and H’m were recorded for the Ingen 54 and Costel varieties. This may be due to the high α-amylase activity of these flours compared to the other varieties of triticale flours (it presents low FN values). As is well known, α-amylase degrades starch and increases the maltose amount from the dough system [43]. Maltose is consumed by yeast with the formation of carbon dioxide leading to an increase in VT and H’m values. However, the H’m value depends not only on the gases formed in the dough during the fermentation process but also due to its ability to retain gases. For example, even if some triticale varieties present high α-amylase activity, they may present a weak gluten network, which cannot retain gases [44], a fact expressed by low VR and CR values and even H’m ones. According to our data, high values of VR have been recorded for Costel and Ingen 54 varieties, which, according to our previous data, also present high wet gluten values. However, these varieties present low CR values, leading us to the conclusion that these dough flours present a low ability to retain the gases released during fermentation.

**Figure 2:**
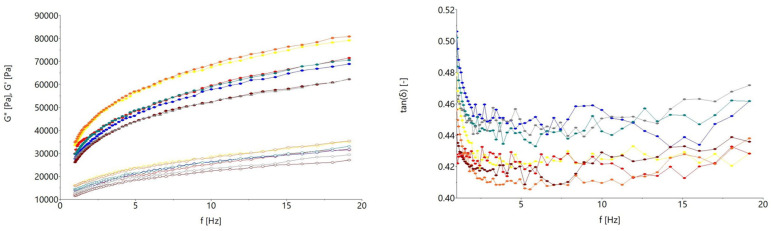
Evaluation with frequency of storage modulus (G′—open symbols), loss modulus (G″—solid symbols) and loss tangent (tan *δ*) for triticale flour dough (-●- Ingen 35 -●- Fanica; -●- Ingen 33; -●- Ingen 40; -●- Costel; -●- Ingen 54; -●- Ingen 93).

The triticale flour dough loss tangent (tan δ), loss modulus (G″) and storage modulus (G′) variation with frequency are shown in Figure 2. As it may be seen, the G″ was lower than G′ in all frequency ranges, indicating that the viscous properties of the triticale dough were less prominent than the elastic ones. The ratio between the G″ and G′ modulus, the tan δ of which is less than 1 for all triticale samples, shows solid-like behavior with values similar to those for dough obtained from wheat flour [45]. The highest G′ and G″ and lowest tan δ values were obtained for the triticale varieties Ingen 40, Costel and Fanica, indicating the fact that these dough samples presented the best viscoelasticity.

### 3.3. Characterization of Triticale Bread Samples

**Figure 3:**
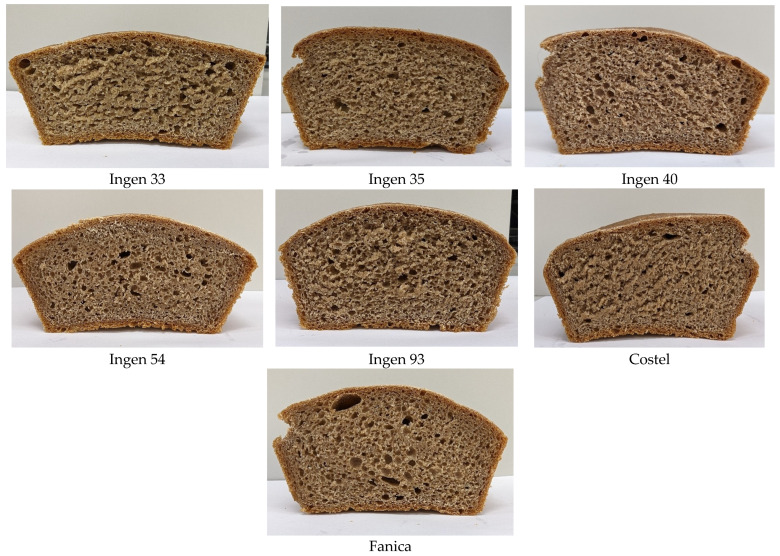
Images of breads obtained from triticale flours varieties used.

Sensory characteristics of triticale bread samples are the most important ones that can influence consumer perception. The sensory and physico-chemical indicators of the triticale bread samples are shown in Table 5, and images of the bread are presented in Figure 3.

During baking, the color, odor, taste and texture of the triticale bread crumb and crust are formed. The sensory evaluation of the triticale bread samples showed that the highest score was given to Ingen 93 (29.85 points), Ingen 54 (29.69 points) and Ingen 35 (28.27 points), and the lowest score was found for Costel (22.34 points); see Table 5. The highest values for the shape and volume of triticale bread were obtained for Ingen 93 (4.0 points) and Ingen 33 (3.87 points). In the case of crust appearance and color, the best results were recorded for Ingen 33, Ingen 54 and Ingen 93. The browning of the triticale bread crust was due to non-enzymatic chemical reactions (caramelization) and the Maillard reaction [46]. Ingen 33 and Costel obtained the lowest scores for the baking degree, state and appearance of bread core: 3.65 points and 3.58 points, respectively. The porosity of the bread core and the pore structure is a characteristic that demonstrates the adequateness of the fermentation process [47]. For this characteristic, the highest scores were accumulated by the triticale bread samples Ingen 54 (5.93 points) and Ingen 93 (5.85 points). It is known that in fermented products such as triticale bread samples, the formation of odor is influenced by the dough preparation process and the ingredients used [48]. Thus, in samples prepared under the same conditions, the odor of the triticale bread essentially did not change, the score being between 3.91 and 4.00 points. Sourdough triticale breads are characterized by higher acidity due to the lactic acid produced during fermentation. The use of *Saccharomyces cerevisiae* yeast in the production of triticale breads led to a pleasant taste, the score being in the range of 5.93–6.0 points. Nevertheless, the total scores for the sensory analysis of the triticale bread samples, with the exception of Costel, were in a range of 25.26–29.85 points, which implies that the products are of very good quality, and Costel was of good quality, accumulating 22.34 points [27].

Physical bread values such as the loaf volume and porosity may be related to many factors: triticale flour composition such as the wet gluten content, its enzymatic activity, dough viscoelasticity, capacity of dough to retain gases during fermentation, etc. [41]. Gluten is a protein formed during dough mixing. It is very important in breadmaking due to its capacity of extension during proofing and baking and also its ability to retain gases formed during fermentation [42]. According to our data, the highest values of the specific volume have been recorded for Ingen 35 and Ingen 33 varieties, who also presented the highest wet gluten values. Additionally, a high α-amylase activity may increase the amount of fermentable sugar and therefore the gases released during fermentation process [34]. During fermentation, the amount of fermentable sugar facilitates the activity of yeast, leading to carbon dioxide formation. However, all our triticale varieties presented high α-amylase activity. If the viscoelastic properties of dough allow dough extension, its low volume presents good values. It may be seen that breads obtained from Ingen 40, Fanica, and Ingen 93 also present high loaf volume values, possibly due to higher dough tenacity and baking strength, which was recorded according to our previous alveograph data. Generally, the bread samples with high loaf volume data also presented higher porosity values. Their values are also affected by the dough gluten content and dough viscoelasticity. However, none of the values obtained indicate bread of a high quality, but this fact was somehow predictable taking into account that the raw material is triticale flour and not wheat flour. The bread with the highest acidity was Ingen 93 followed by Ingen 33, Ingen 54 and Ingen 35. The lowest acidity was recorded for the bread sample obtained from triticale flour variety Ingen 40.

The textural parameters of triticale bread are shown in Table 6. The hardness value presented significant differences (*p* < 0.05) among bread samples. This may be due to the protein content of the bread samples. The bread sample obtained from the triticale flour with the highest protein content, Ingen 35, presented the lowest hardness value, whereas the bread sample obtained from Ingen 40triticale flour with the lowest protein content presented the highest hardness value. These triticale flours may vary in their protein–starch–pentosant complex, which can affect bread hardness. Additionally, a bread sample with weaker gluten is less elastic, decreasing the hardness value of bread samples [49]. Gumminess is a product of cohesiveness and hardness while chewiness is a product of springiness and gumminess [50]. Therefore, increased chewiness and gumminess values will lead to an increase in springiness, cohesiveness and hardness. The bread samples obtained from the triticale flours with the highest gluten content, Ingen 35 and Ingen 33, presented the lowest gumminess values. These data are in agreement with those reported by others [51,52], who also concluded that an increase in gluten content would lead to lower gumminess, hardness and chewiness values. More gluten could enhance protein stability from the dough system and the water mobility in triticale bread, resulting in products of good quality. Higher resilience corresponds to stronger doughs. The triticale dough obtained from the Ingen 54 variety presented the highest stability and dough development time and therefore the highest resilience value. According to Guo et al. [51], cohesiveness may reflect the external damage resistance and internal compactness of bread. Its value may increase with the increased gluten content [52], but it may vary due to the gluten strength or processing methods [50].

### 3.4. Relationships between Flour, Dough and Bread Characteristics

**Figure 4:**
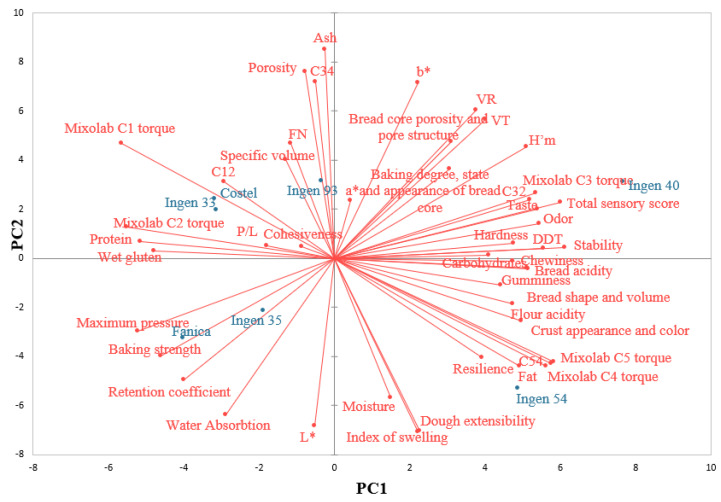
Principal component analysis: C12, difference in torques C1 and C2; C32, difference in torques C3 and C2; C34, difference in torques C3 and C4; C54, difference in torques C5 and C4; H’m, maximum height of gaseous production; VT, total CO_2_ volume production; VR, volume of the gas retained in the dough at the end of the test; CR, retention coefficient.

The relationships between flour, dough and bread characteristics through principal component analysis (PCA) are shown in Figure 4. The PC1 and PC2 components accounted for 37.11% and 22.02% of the total variance. The cohesiveness and P/L ratio do not present a high contribution to the data variation according to their position on the PCA, close to the center. Contrarily, highly significant (*p* < 0.05) correlations were obtained between different parameters, a fact also suggested by their closeness on the graph, for example: protein and C12 (r = 0.807); fat and C4 (r = 0.852), C5 (r = 0.846), C54 (r = 0.840), and resilience (r = 0.859); WA and P (r = 0.822) and W (r = 0.833); stability and DDT (r = 0.987) and H’m (r = 0.765); C3 and C32 (r = 0.993) and bread shape and volume (r = 0.842); C4 and C54 (r = 0.994) and crust appearance and color (r = 0.800); C5 and C54 (r = 0.999); crust appearance and C4 (r = 0.800), C5 (r = 0.8215), C54 (r = 0.836), and bread shape and volume (r = 0.831); bread shape and volume and crust appearance and color (r = 0.828); total sensory score and C3 (r = 0.876) and C32 (r = 0.821); baking degree state and appearance (r = 0.786), odor (r = 0.972), and taste (r = 0.947); hardness and gumminess (r = 0.912) and chewiness (r = 0.910). The PC1 is closely associated with protein, wet gluten, Mixolab C2 torque, carbohydrates, chewiness, DDT, stability, hardness, bread acidity, and gumminess whereas PC2 is closely associated with moisture, index of swelling, L*, ash, C34, porosity, FN, specific volume, a*, b*, etc. According to the PCA graph, the triticale varieties that are most closely to each other are Ingen 93, Costel, and Ingen 33, which are situated at the top left of the graph, and Fanica and Ingen 35, which are positioned at the bottom left of the PCA. Both PCA components distinguish Ingen 54 from Ingen 33, Costel, Ingen 93 and Ingen 40 from Fanica and Ingen 35, indicating an inverse correlation between these varieties. The first component, PC1, distinguishes Ingen 33, Costel, Ingen 93, and Ingen 40 from Fanica, Ingen 35, and Ingen 54, whereas the second component, PC2, distinguishes Ingen 40 and Ingen 54 from Fanica, Ingen 35, Ingen 33, Costel, and Ingen 93 triticale varieties. Regarding triticale variety correlations with their analyzed characteristics, it seems that the Fanica and Ingen 35 varieties are more related to the alveograph data and water absorption value; Ingen 33, Costel, Fanica with P/L, Mixolab C2 torque, C12, specific volume, FN, and Ingen 40 are related to many sensory characteristics such as the total sensory score, odor, taste and Ingen 54 to Mixolab C5, C4 and C54 parameters, fat, resilience, and dough extensibility.

## 4. Conclusions

Significant variations in flour, dough and bread quality characteristics were found among the seven investigated triticale cultivars from the Republic of Moldova. Except for the wet gluten content, all physico-chemical parameters analyzed indicate that triticale flour is appropriate for use in breadmaking. Ingen 93, Ingen 54 and Ingen 40 presented lower values for wet gluten content, which may indicate some difficulties in obtaining bread from these types of triticale flours. The Mixolab data indicate high stability and a long development time for Ingen 40 and Ingen 54 triticale varieties, high viscosity during heating for Ingen 40, and lower retrogradation of the starch for Ingen 33, Costel and Fanica, which may indicate a longer shelf life for the bread samples obtained from these varieties. Regarding the falling number values, all triticale flour samples presented low values, which indicates high α-amylase activity. According to the triticale dough behavior during extension, all the samples presented low extensibility and high tenacity values. During fermentation, even if some samples presented high gas production, they were not very well retained by the dough structure, the highest retention coefficient being recorded for the Ingen 35 sample. According to the dynamic rheometer data, the best viscoelasticity values were obtained for the Ingen 40, Costel and Fanica triticale varieties. From all the triticale flours, bread was obtained. The highest loaf volume was obtained for Ingen 33, followed by Ingen 35, for which the highest porosity was recorded. The sensory analysis showed that the total scores of the triticale bread samples (Ingen 33, Ingen 35, Ingen 40 Ingen 54, Ingen 93 and Fanica) were in the range of 25.26–29.85 points, which implies that the products are of very good quality, and Costel was of good quality, accumulating 22.34 points. All the data obtained indicate that the triticale flours analyzed within this study may be sustainable for breadmaking. However, the best results were obtained for Ingen 33 and Ingen 35, which we recommend for bakery product production.

## Figures and Tables

**Table 1:** Physico-chemical parameters of triticale flour.

Parameters	Ingen 35	Ingen 33	Ingen 93	Ingen 54	Ingen 40	Fanica	Costel
Moisture (%)	12.2 ± 0.10 ^ab^	12.2 ± 0.05 ^a,b^	12.1 ± 0.10 ^a^	12.4 ± 0.10 ^b^	12.3 ± 0.10 ^a,b^	12.4 ± 0.10 ^b^	12.3 ± 0.1 ^a,b^
Ash (%)	1.6 ± 0.10 ^d^	1.7 ± 0.1 ^d^	1.8 ± 0.10 ^d^	1.5 ± 0.10 ^b^	1.7 ± 0.10 ^a^	1.5 ± 0.10 ^b^	1.7 ± 0.1 ^c^
Protein (%)	14.8 ± 0.10 ^f^	14.6 ± 0.1 ^g^	14.6 ± 0.10 ^b^	13.7 ± 0.10 ^c^	13.1 ± 0.10 ^a^	13.9 ± 0.10 ^d^	14.2 ± 0.1 ^e^
Wet gluten (%)	26.14 ± 0.10 ^b^	27.49 ± 0.2 ^b,c^	18.55 ± 0.10 ^b,c^	19.6 ± 0.10 ^d^	18.49 ± 0.10 ^c^	21.63 ± 0.10 ^b^	25.61 ± 0.1 ^a^
Fat (%)	1.3 ± 0.05 ^a,b^	1.36 ± 0.1 ^b^	1.35 ± 0.10 ^a,b^	1.7 ± 0.05 ^a^	1.46 ± 0.05 ^a,b^	1.31 ± 0.10 ^a^	1.07 ± 0.05 ^a,b^
Carbohydrates (%)	70.1 ± 0.05 ^a^	70.14 ± 0.10 ^a^	70.15 ± 0.10 ^a^	70.7 ± 0.10 ^b^	71.44 ± 0.10 ^c^	70.89 ± 0.10 ^b^	70.7 ± 0.05 ^b^
Acidity (grade)	2.9 ± 0.10 ^b^	2.7 ± 0.10 ^a,b^	2.7 ± 0.05 ^a,b^	3.2 ± 0.05 ^c^	3.9 ± 0.10 ^d^	3.4 ± 0.10 ^c^	2.5 ± 0.10 ^a^
L*	81.95 ± 1.45 ^c^	81.47 ± 0.73 ^b^	82.13 ± 0.69 ^d^	82.62 ± 0.47 ^e^	81.08 ± 0.56 ^a^	82.10 ± 0.76 ^d^	81.48 ± 0.59 ^b^
a*	2.01 ± 0.03 ^a^	2.11 ± 0.05 ^b^	2.30 ± 0.02 ^d^	2.23 ± 0.04 ^c^	2.39 ± 0.02 ^e^	2.43 ± 0.01 ^f^	2.51 ± 0.03 ^g^
b*	11.49 ± 0.15 ^d^	11.85 ± 0.13 ^e^	12.18 ± 0.09 ^f^	10.79 ± 0.11 ^a^	12.39 ± 0.13 ^g^	10.95 ± 0.09 ^b^	11.14 ± 0.06 ^c^

The results are mean ± standard deviation. Means (*n* = 3 for chemical data, *n* = 10 for color data) followed by the same letter within a column are not significantly different (*p* < 0.05). L*—darkness/brightness, a*—shade of red/green, b*—shade of blue/yellow.

**Table 2:** Mixing and pasting parameters of triticale flour dough.

Parameters	Ingen 35	Ingen 33	Ingen 93	Ingen 54	Ingen 40	Fanica	Costel
WA (%)	59.5 ± 0.05 ^b,c^	60.1 ± 0.1 ^d^	59.0 ± 0.2 ^b^	60.8 ± 0.2 ^e^	58.8 ± 0.2 ^a^	61.7 ± 0.1 ^f^	60.1 ± 0.1 ^d^
ST (min)	2.1 ± 0.01 ^b^	1.9 ± 0.01 ^a^	2.1 ± 0.02 ^b^	2.7 ± 0.01 ^d^	3.8 ± 0.01 ^e^	2.6 ± 0.02 ^c,d^	2.3 ± 0.01 ^b,c^
DDT (min)	2.63 ± 0.02 ^b^	2.48 ± 0.01 ^a^	2.70 ± 0.02 ^b^	3.23 ± 0.02 ^d^	4.08 ± 0.04 ^e^	2.9 ± 0.02 ^c^	2.70 ± 0.01 ^b^
C1 (N∙m)	1.121 ± 0.01 ^c^	1.145 ± 0.02 ^e^	1.134 ± 0.01 ^d^	1.082 ± 0.02 ^a^	1.097 ± 0.01 ^b^	1.119 ± 0.01 ^c^	1.146 ± 0.02 ^e^
C2 (N∙m)	0.363 ± 0.05 ^b^	0.374 ± 0.02 ^c^	0.371 ± 0.01 ^c^	0.358 ± 0.02 ^a,b^	0.390 ± 0.03 ^e^	0.419 ± 0.02 ^f^	0.383 ± 0.01 ^d^
C3 (N∙m)	1.51 ± 0.14 ^c^	1.58 ± 0.17 ^d^	1.57 ± 0.12 ^d^	1.58 ± 0.14 ^d^	1.71 ± 0.17 ^e^	1.31 ± 0.17 ^a^	1.36 ± 0.12 ^b^
C4 (N∙m)	0.41 ± 0.07 ^c^	0.24 ± 0.09 ^a^	0.35 ± 0.04 ^b^	0.65 ± 0.04 ^e^	0.47 ± 0.09 ^d^	0.26 ± 0.04 ^a^	0.24 ± 0.02 ^a^
C5 (N∙m)	0.82 ± 0.02 ^c^	0.44 ± 0.01 ^a^	0.65 ± 0.02 ^b^	1.34 ± 0.01 ^e^	0.98 ± 0.01 ^d^	0.48 ± 0.01 ^a^	0.44 ± 0.01 ^a^
C12 (N∙m)	0.758 ± 0.04 ^d^	0.771 ± 0.01 ^f^	0.763 ± 0.02 ^e^	0.734 ± 0.04 ^b^	0.707 ± 0.01 ^b^	0.700 ± 0.01 ^a^	0.763 ± 0.02 ^e^
C32 (N∙m)	1.147 ± 0.11 ^c^	1.206 ± 0.06 ^d^	1.199 ± 0.09 ^d^	1.222 ± 0.11 ^e^	1.320 ± 0.09 ^f^	0.891 ± 0.06 ^a^	0.977 ± 0.11 ^b^
C34 (N∙m)	1.100 ± 0.05 ^b,c^	1.340 ± 0.12 ^e^	1.220 ± 0.13 ^d^	0.930 ± 0.10 ^a^	1.240 ± 0.05 ^d^	1.050 ± 0.10 ^b^	1.120 ± 0.12 ^c^
C54 (N∙m)	0.410 ± 0.01 ^c^	0.200 ± 0.02 ^a^	0.300 ± 0.01 ^b^	0.690 ± 0.02 ^e^	0.510 ± 0.01 ^d^	0.220 ± 0.01 ^a^	0.200 ± 0.01 ^a^
FN (s)	63 ± 1.52 ^a^	70 ± 0.57 ^d^	67 ± 1.00 ^b^	63 ± 0.57 ^a^	67 ± 1.52 ^b,c^	67 ± 1.00 ^c^	64 ± 0.57 ^a,b^

Mixolab values: WA, water absorption; ST, stability, DDT, dough development time; C1–C5, torques corresponding to stages 1–5 of the Mixolab curve; C12, difference in torques C1 and C2; C32, difference in torques C3 and C2; C34, difference in torques C3 and C4; C54, difference in torques C5 and C4. ^a–f^, means (*n* = 3) followed by the same letter within a column are not significantly different (*p* < 0.05).

**Table 3:** Alveograph data of triticale dough.

Triticale Samples	P (mm)	L (mm)	G (mm)	W (10^−4^ J)	P/L
Ingen 35	98 ± 1.00 ^b,c^	29 ± 1.15 ^d^	12.0 ± 0.28 ^d^	96 ± 2.00 ^c^	3.38 ± 0.02 ^b^
Ingen 40	117 ± 2.5 ^d^	27 ± 1.00 ^c^	11.5 ± 0.30 ^c^	123 ± 2.00 ^e^	4.33 ± 0.03 ^d^
Ingen 33	97 ± 1.00 ^b^	24 ± 1.00 ^a^	10.9 ± 0.15 ^a^	88 ± 1.00 ^b^	4.04 ± 0.12 ^c^
Ingen 93	100 ± 2.00 ^b,c^	31 ± 2.00 ^e^	12.4 ± 2.00 ^e^	103 ± 2.00 ^d^	3.23 ± 0.04 ^a^
Ingen 54	85 ± 2.00 ^a^	26 ± 1.00 ^b^	11.3 ± 0.46 ^b,c^	76 ± 2.00 ^a^	3.27 ± 0.03 ^a^
Fanica	127 ± 1.00 ^e^	26 ± 1.00 ^b^	11.3 ± 0.15 ^b,c^	127 ± 3.00 ^e,f^	4.88 ± 0.18 ^e^
Costel	104 ± 1.00 ^c^	24 ± 1.00 ^a^	10.9 ± 0.15 ^a^	97 ± 1.00 ^c^	4.33 ± 0.02 ^d^

P, maximum pressure; L, dough extensibility; G, index of swelling; W, baking strength; P/L, configuration ratio of the alveograph curve. ^a–f^, mean values (*n* = 3) in the same column followed by different letters are significantly different (*p* < 0.05).

**Table 4:** Rheofermentometer data of triticale dough.

Dough Samples	H’m (mm)	VT (mL)	VR (mL)	CR (%)
Ingen 35	55.6 ± 0.6 ^a,b^	1187 ± 3 ^a^	989 ± 1 ^a^	83.3 ± 0.1 ^d,e^
Ingen 40	54.8 ± 0.2 ^a^	1216 ± 5 ^b^	1006 ± 3 ^c^	82.7 ± 0.2 ^d^
Ingen 33	58.8 ± 0.3 ^d^	1272 ± 3 ^c^	1029 ± 2 ^d^	80.9 ± 0.1 ^c^
Ingen 93	57.9 ± 0.1 ^c,d^	1232 ± 1 ^b^	1002 ± 3 ^b,c^	81.3 ± 0.1 ^c^
Ingen 54	64.1 ± 0.2 ^f^	1375 ± 2 ^e^	1071 ± 2 ^f^	77.9 ± 0.01 ^a^
Fanica	55.0 ± 0.1 ^a^	1223 ± 3 ^b^	1011 ± 1 ^c^	82.7 ± 0.3 ^d^
Costel	59.9 ± 0.1 ^e^	1307 ± 4 ^d^	1032 ± 3 ^d^	78.9 ± 0.10 ^a,b^

H’m, maximum height of gaseous production; VT, total CO_2_ volume production; VR, volume of the gas retained in the dough at the end of the test; CR, retention coefficient; ^a–f^, mean values (*n* = 3) in the same column followed by different letters are significantly different (*p* < 0.05).

**Table 5:** Sensory characteristics and physico-chemical indicators of triticale bread samples.

Characteristics/Indicators	Triticale Bread Samples						
	Ingen 33	Ingen 35	Ingen 40	Ingen 54	Ingen 93	Costel	Fanica
Sensory characteristics							
Total sensory score	25.26 ± 0.04 ^b^	28.27 ± 0.16 ^e^	26.81 ± 0.21 ^d^	29.69 ± 0.08 ^f^	29.85 ± 0.03 ^f^	22.34 ± 0.13 ^a^	25.80 ± 0.11 ^c^
Bread shape and volume	3.87 ± 0.06 ^e^	3.56 ± 0.08 ^d^	2.74 ± 0.04 ^c^	3.76 ± 0.05 ^e^	4.00 ± 0.0 ^f^	2.11 ± 0.03 ^b^	1.95 ± 0.04 ^a^
Crust appearance and color	4.00 ± 0.0 ^d^	2.96 ± 0.03 ^c^	2.49 ± 0.04 ^b^	4.00 ± 0.0 ^d^	4.00 ± 0.0 ^d^	2.14 ± 0.06 ^a^	2.21 ± 0.03 ^a^
Baking degree, state and appearance of bread core	3.65 ± 0.08 ^a^	6.00 ± 0.0 ^b^	6.00 ± 0.0 ^b^	6.00 ± 0.0 ^b^	6.00 ± 0.0 ^b^	3.58 ± 0.04 ^a^	6.00 ± 0.0 ^b^
Bread core porosity and pore structure	3.85 ± 0.06 ^a^	5.81 ± 0.03 ^c,d^	5.64 ± 0.05 ^c^	5.93 ± 0.04 ^d^	5.85 ± 0.03 ^c,d^	4.65 ± 0.07 ^b^	5.74 ± 0.02 ^c^
Odor	3.95 ± 0.01 ^b,c^	3.98 ± 0.0 ^d^	3.96 ± 0.01 ^b,c,d^	4.00 ± 0.0 ^e^	4.00 ± 0.0 ^e^	3.91 ± 0.0 ^a^	3.97 ± 0.01 ^c,d^
Taste	5.94 ± 0.01 ^a,b,c^	5.96 ± 0.0 ^d^	5.98 ± 0.0 ^e^	6.00 ± 0.0 ^f^	6.00 ± 0.0 ^f^	5.95 ± 0.01 ^c,d^	5.93 ± 0.01 ^a,b^
Physico-chemical indicators							
Specific volume (cm^3^/100 g)	265 ± 0.74 ^e^	259 ± 0.75 ^d^	243 ± 0.75 ^b^	229 ± 2.15 ^a^	248 ± 2.27 ^c^	228 ± 2.15 ^a^	246 ± 0.75 ^c^
Porosity (%)	55.8 ± 0.20 ^c^	62.4 ± 0.36 ^g^	59.5 ± 0.10 ^f^	52.6 ± 0.20 ^b^	57.7 ± 0.36 ^e^	51.3 ± 0.10 ^a^	56.6 ± 0.10 ^d^
Acidity (grade)	4.5 ± 0.10 ^d^	4.2 ± 0.10 ^b,c^	3.8 ± 0.20 ^a^	4.3 ± 0.10 ^c,d^	4.9 ± 0.10 ^e^	4.0 ± 0.10 ^a,b^	4.0 ± 0.10 ^a,b^

^a–g^, mean values (*n* = 3) in the same column followed by different letters are significantly different (*p* < 0.05).

**Table 6:** Textural parameters of triticale bread.

Bread Samples	Hardness (N)	Gumminess (N)	Chewiness (N)	Cohesiveness (Adimensional)	Resilience (Adimensional)
Ingen 35	29.20 ± 0.01 ^b^	19.37 ± 0.01 ^a,b^	19.31 ± 0.01 ^a^	0.66 ± 0.01 ^a,b^	0.36 ± 0.01 ^a,b^
Ingen 33	23.45 ± 0.02 ^a^	19.17 ± 0.01 ^a,b^	19.14 ± 0.01 ^a^	0.82 ± 0.01 ^e^	0.42 ± 0.01 ^d^
Ingen 93	41.29 ± 0.01 ^f^	30.17 ± 0.02 ^e^	30.18 ± 0.02 ^e^	0.73 ± 0.01 ^d^	0.37 ± 0.01 ^b^
Ingen 54	37.80 ± 0.02 ^e^	30.77 ± 0.01 ^e,f^	30.83 ± 0.01 ^f^	0.81 ± 0.01 ^e^	0.51 ± 0.00 ^e^
Ingen 40	43.57 ± 0.01 ^g^	29.51 ± 0.01 ^e^	29.51 ± 0.01 ^e^	0.68 ± 0.01 ^a,b,c^	0.40 ± 0.01 ^c^
Fanica	35.81 ± 0.01 ^d^	24.20 ± 0.02 ^d^	24.15 ± 0.02 ^d^	0.69 ± 0.01 ^c^	0.36 ± 0.00 ^a^
Costel	31.51 ± 0.01 ^c^	21.90 ± 0.01 ^b,c^	21.81 ± 0.01 ^c^	0.81 ± 0.01 ^a,b^	0.36 ± 0.00 ^a^

^a–g^, mean (*n* = 3) values in the same column followed by different letters are significantly different (*p* < 0.05).

## Data Availability

The original contributions presented in the study are included in the article; further inquiries can be directed to the corresponding author.
